# Laparoscopic Pyelolithotomy for treating urolithiasis in ectopic pelvic kidneys

**DOI:** 10.1590/S1677-5538.IBJU.2023.0148

**Published:** 2023-05-15

**Authors:** Ioannis Kartalas Goumas, Elena Tondelli, Luigi Bevilacqua, Isabella Oliva, Luca Orecchia, Marcelo Langer Wroclawski, Guido Giusti, Silvia Proietti, Roberto Miano, Eugenio Ventimiglia

**Affiliations:** 1 Istituto Clinico Beato Matteo Division of Urology Vigevano Italy Division of Urology, Istituto Clinico Beato Matteo, Vigevano, Italy; 2 University of Rome Tor Vergata Division of Urology Department of Surgical Sciences Rome Italy Division of Urology, Department of Surgical Sciences, University of Rome Tor Vergata, Rome, Italy; 3 Hospital Israelita Albert Einstein São Paulo SP Brasi Hospital Israelita Albert Einstein, São Paulo, SP, Brasi; 4 IRCCS San Raffaele Hospital Department of Urology Milan Italy Department of Urology, IRCCS San Raffaele Hospital, Milan, Italy; 5 Urological Research Institute Division of Experimental Oncology Unit of Urology Mila Italy Division of Experimental Oncology/Unit of Urology, Urological Research Institute, IRCCS Ospedale San Raffaele, Milan, Italy

## Abstract

**Introduction:**

The management of urolithiasis ectopic pelvic kidneys (EPK) can be challenging because of the aberrant anatomy (1–4). We demonstrate the step-by-step technique of the laparoscopic approach for treating urolithiasis in EPK.

**Patients and methods:**

Three men with EPK (2 left, 1 right) underwent laparoscopic pyelolithotomy through a transperitoneal approach. After establishing the pneumoperitoneum, the parietal peritoneum was opened at the parietal colic sulcus and the bowel displaced medially. The kidney was identified in the retroperitoneum and the renal pelvis exposed after removal of the perirenal adipose tissue. The renal pelvis was opened, and the stones were identified and retrieved with forceps in 2 cases and with a flexible nephroscope in 1 case. The renal pelvis was closed with a 3/0 running barbed suture. A DJ stent was placed in all patients.

**Results:**

For the first time, a laparoscopic technique for treating stones in the ectopic kidney is demonstrated in detail. Mean patient age was 52.6 years (44-58). The mean stone size was 22.3 mm (20-24 mm). Stones were in the renal pelvis in 2 cases and in the inferior calyx in 1 case. Mean operative time was 146 minutes (135-155 min). Mean estimated blood loss was 116 ml (60-140 ml). No complications were observed. The mean hospital stay was 3 days. The DJ stents were removed after 3 weeks. All patients were stone free at the postoperative CT scan with a mean follow-up of 3.3 months (1-6 months).

**Conclusions:**

Laparoscopic pyelolithotomy can be an effective and reproducible minimally invasive technique for treating urolithiasis in EPK.

**Available at:** http://www.intbrazjurol.com.br/video-section/20230148_goumas_et_al

**Int Braz J Urol. 2023; 49 (Video #11): 646-7**
